# Red mason bees cannot compete with honey bees for floral resources in a cage experiment

**DOI:** 10.1002/ece3.1762

**Published:** 2015-10-16

**Authors:** Anika Hudewenz, Alexandra‐Maria Klein

**Affiliations:** ^1^ Institute of EcologyEcosystem FunctionsLeuphana University of LüneburgScharnhorststr. 1D‐21335LüneburgGermany; ^2^ Faculty of Environment and Natural ResourcesNature Conservation and Landscape EcologyUniversity of FreiburgTennenbacher Str. 4D‐79106FreiburgGermany

**Keywords:** Cage experiment, competitive interactions, niche breadth, reproduction, resource overlap

## Abstract

Intensive beekeeping to mitigate crop pollination deficits and habitat loss may cause interspecific competition between bees. Studies show negative correlations between flower visitation of honey bees (*Apis mellifera*) and wild bees, but effects on the reproduction of wild bees were not proven. Likely reasons are that honey bees can hardly be excluded from controls and wild bee nests are generally difficult to detect in field experiments. The goal of this study was to investigate whether red mason bees (*Osmia bicornis*) compete with honey bees in cages in order to compare the reproduction of red mason bees under different honey bee densities. Three treatments were applied, each replicated in four cages of 18 m³ with 38 red mason bees in all treatments and 0, 100, and 300 honey bees per treatment with 10–20% being foragers. Within the cages, the flower visitation and interspecific displacements from flowers were observed. Niche breadths and resource overlaps of both bee species were calculated, and the reproduction of red mason bees was measured. Red mason bees visited fewer flowers when honey bees were present. Niche breadth of red mason bees decreased with increasing honey bee density while resource overlaps remained constant. The reproduction of red mason bees decreased in cages with honey bees. In conclusion, our experimental results show that in small and isolated flower patches, wild bees can temporarily suffer from competition with honey bees. Further research should aim to test for competition on small and isolated flower patches in real landscapes.

## Introduction

The growing demand for pollination in agriculture has raised attention (Aizen and Harder 2009) and led to a widespread use of honey bee colonies placed in fields to provide pollination service (e.g., almonds in California). However, the massive expansion of flowering crops may enhance abundances of honey bees (Holzschuh et al. 2011), which could be competitors for flower resources in surrounding natural habitat patches. In particular, before or after the peak of crop flowering, honey bees might switch to forage on flowers of surrounding natural habitats. Despite recent concern over the threats posed to honey bees by diseases, the worldwide number of domesticated honey bee colonies continues to increase (Aizen and Harder 2009). In contrast, wild bees are declining (Biesmeijer et al. 2006; Potts et al. 2010). Unlike honey bees, wild bee losses cannot be easily monitored.

When multiple bee species share a limited amount of the same resources, competition for these resources may occur (van Veen et al. 2006). Interspecific competition for floral resources may result in the exploitation of food resources by the stronger competitor and a reduced food supply for the weaker competitor. The European honey bee (*Apis mellifera* L.) is considered to be a strong competitor because it is highly efficient in exploiting floral resources. It is a social and generalist bee species visiting a broad range of different plant species (Crane 1990), and workers can communicate the location of flower‐rich and abundant resource patches to each other. Conservationists are concerned that honey bees exploit floral resources and outcompete unmanaged wild bees (e.g., Evertz 1995; Thomson 2004). If honey bees are abundant and exploit floral resources, the number of flower visits of wild bees might decrease, which in turn leads to a reduced food supply for the larvae of wild bees and reduced reproductive success. However, if a wild bee species is a generalist and can find food resources on different plant species, floral resource exploitation by honey bees could lead to a niche shift of the wild bee (Walther‐Hellwig et al. 2006). The reproduction of the wild bee might then not be affected, but this can be reflected by a reduced niche breadth of the wild bee and a reduced resource overlap with honey bees. In addition to competition due to resource exploitation, studies have shown interspecific encounters between bee species leading to the displacement of one bee by another from a flower (Pinkus‐Rendon et al. 2005; Rogers et al. 2013). This can modify foraging behavior and food utilization of the displaced bees (Rogers et al. 2013). If honey bees actively displace wild bees from flowers, then higher disturbance and reduced food supply of the wild bees may occur.

This study investigates whether the generalist and solitary red mason bee, *Osmia bicornis* L., is able to compete with social honey bees in cages that represented small and isolated flower‐rich habitat patches. The red mason bee was chosen because it is native to central Europe and it is one of the most common wild bee species in Germany. Because it is a generalist, its resource use can be assumed to overlap with the resource use of the generalist honey bee. In most field experiments, the densities of honey bees are artificially high due to the presence of domesticated colonies for honey and wax production and pollination, such that sites lacking honey bees are rare. This could be one reason why earlier studies did not show a reduced reproductive success of wild bees caused by competition with honey bees. Therefore, we decided to conduct a cage experiment in which honey bees can be excluded from control treatments. Another advantage of a cage experiment is that it allows for the investigation into the reproductive success of all exposed wild bees. In field studies, solitary bee nests can also be located outside of the studied site and remain uncounted. In addition to control treatments without honey bees, we chose two different treatments comprising two different honey bee/wild bee ratios. One of the respective honey bee/wild bee ratio included fewer honey bees than usually found in field experiments in Germany, while the other ratio included more honey bee individuals and was close to the ratio found in grasslands (Hudewenz et al. 2012) but lower than ratios found in a heathland with active beekeeping (Hudewenz and Klein 2013) (Table S1).

We hypothesize that

1. The number of red mason bee flower visits decreases with increasing honey bee flower visits and with increasing number of honey bee individuals.
2. Honey bees displace red mason bees actively from flowers.
3. The niche breadth of honey bees increases with increasing number of honey bee individuals while the niche breadth of the red mason bee decreases.
4. The resource overlap of red mason bees and honey bees increases with increasing number of honey bee individuals.
5. Red mason bees produce fewer offspring when honey bees are abundant.

## Materials and Methods

### Study area and study design

We conducted a cage experiment in 2013 on an experimental field site in Lüneburg, Germany, which is owned by the Leuphana University of Lüneburg. In November 2012, native soil (composted earth from “GFA Lüneburg”) was distributed on twelve marked 3 × 3 m patches that were established to become covered by gauze cages in the next year. For all patches, the same soil was used to standardize growth conditions for all plants across all cages. The bare soil was covered with weed control mats (150 g/m^2^) to reduce the growth of nontarget plants. On 07 April 2013, a mixture of eleven different flowering plant species “Tübinger Mischung” (ordered at www.nisthabitate.info, Table S2) was sown on each of the twelve marked patches. Additionally, five nontarget plant species (weeds) were growing and flowering in the cages (Table S2). When the sown plants started to flower (06‐Jun‐2013), the gauze cages were set up. Each gauze cage was 3 × 3×2 m (length × width × height; equally to 18 m³), with a mesh size of 240 per square inch (insect net “Terrazzo”, ordered at www.mehari-offroad.de). All plots were regularly watered before and during the experiment. Flowers produced nectar and pollen during the experiment.

The red mason bee is solitary, meaning that females build their nests on their own without sharing the brood care. They nest in hollow stems and holes in walls and can be readily caught in trap nests. Each nest is comprised of 1–14 brood cells in which eggs are laid individually and provisioned with pollen mixed with nectar.

The European honey bee is eusocial and creates colonies of an average of 40,000 individuals. To reduce the number of individuals per colony, mating colonies were used, which are composed of one queen and 100–300 worker bees. Only 10–20% of the worker bees were foraging on flowers (forager bees). As the honey bees were placed in the mating colonies shortly before the start of the experiment, the feed stock (honey) inside the colonies was still lacking. Therefore, the honey bees were fed with sugar patties throughout the experiment. Honey bee worker numbers were estimated visually before and at the end of the experiment. During the experiment, the number of bee workers increased in five of the eight colonies, while in two colonies the number remained constant, and in only one colony, the number of workers declined. No anomalies in the foraging behavior of red mason bees and honey bees were observed in the cages compared to foraging in the field (e.g., bees were not flying into the gauze or hanging from the top of the gauze).

On 07 June 2013, 38 newly emerged individuals of the red mason bee (10 females and 28 males) were introduced to each cage as we found this sex ratio for this bee within trap nest in different field experiments in Germany. During this time, we frequently observed the red mason bees mating. On 11 Jun 2013, honey bee mating colonies containing 100 individuals were placed in four of the cages, and mating colonies containing 300 honey bee individuals were placed in the other four cages. The remaining four cages contained only the red mason bee individuals and no honey bees.

In each cage, 500 mL of sand was placed as nest building material for red mason bees and a bowl of water for nest or colony building for the two species. The experiment continued until 17 July 2013, when the cages were removed.

### Flower observations

Throughout the experiment, flower observations were conducted to study the resource use of both bee species starting on 17 June 2013 until 12 July 2013. Observations were carried out between 8:00 and 18:00 when temperatures exceeded 17°C and there was no rain. During observations, the number of flower visits by honey bees and red mason bees per plant species was counted. Additionally, the number of interactions in which a bee species forced an interspecific bee to leave the recently visited flower was counted. In each cage, the flowers on an area of 1.5 × 1.5 m were observed for 10 min per observation interval. As not all sown plant species flowered at the same time, the flowers of 2–4 different plant species were observed during one observation interval.

A total of 14 observation intervals were conducted resulting in a total observation time of 140 min per cage. For each observation, the flower cover was estimated in the cage as the proportion of open flowers to the size of the cages. Additionally, the number of currently flowering plant species was counted.

### Trap nests

In each cage, two empty trap nests were exposed on one wooden post (at 1.3 m height) in the beginning of the cage experiment. Trap nests consisted of a plastic tube of 22 cm length and 10.5 cm diameter, filled with around 100 reed internodes of different diameters with the majority ranging from 5 to 7 mm as these diameters are commonly used by red mason bees (Free and Williams 1970). When the cages were removed, the trap nests were collected and all reed internodes were checked for completed nests. Subsequently, the number of nests and brood cells was counted.

### Statistical analyses

All statistical analyses were conducted using R 2.15.2 for Windows (R Development Core Team 2012). To test the first and the fifth hypotheses, the following response variables were analyzed using generalized linear mixed models: (1) number of honey bee flower visits per observation interval, (2) number of red mason bee flower visits per observation interval, and (3) number of brood cells of the red mason bee per trap nest (glmer function in package “lme4” (Bates and Maechler 2010)). All models were fitted with a Poisson error distribution and contained the number of the flight cage as a random effect. For response variable (1), the explanatory variables, number of honey bee individuals per cage and flower cover, were tested. For the response variable (2), the three explanatory variables, number of honey bee individuals per cage, number of honey bee flower visits, flower cover and number of flowering plant species, were tested. The tested explanatory variable for (3) was the number of honey bee individuals. Initial models did not contain explanatory variables. The decision if explanatory variables need to be included in the models and in which order they have to be included was chosen using the corrected Akaike information criterion (AICc). Models were manually compared step by step. Those variables that were not included in the model because of a higher AICc were assumed not to be explanatory for the response variable. For the correlated explanatory variables, number of honey bee individuals and number of honey bee flower visits, separate models were established to avoid collinearity.

To test the second hypothesis, the niche breadth of red mason bees and of honey bees per observation interval was calculated. We used the measure proposed by Smith (1982) because it includes the abundance of resources, which was estimated as the flower cover of the different plant species in our study. The Smith's measure of niche breadth is the sum of the roots of the proportions of flower visits on single plant species times the flower cover. We compared the niche breadths of each species per treatment using ANOVAs type III.

For the third hypothesis, the percentage resource overlap (Renkonen 1938) and the Hurlbert's Index for resource overlap (Hurlbert 1978) were calculated per observation interval. The percentage overlap is the sum of the shared proportion the single resource is used of the total resources used by both species. In contrast to the percentage overlap, the Hurlbert's Index also takes into account the abundance of available resources. The percentage overlap and the Hurlbert's Index were compared between treatments using ANOVAs type III.

## Results

### Flower observations

Of all flower visits, 59% were conducted by honey bees and 41% by red mason bees. Both species visited mainly the three most abundant flowering plant species: mustard (*Sinapis arvensis* L.), lacy phacelia (*Phacelia tanacetifolia* benth), and buckwheat (*Fagopyrum esculentum* mill), with fewer visits to the species that flowered in lower abundances (Table S2). Honey bees most frequently visited lacy phacelia (9227 visits) followed by mustard (4485 visits) and buckwheat (592 visits). Red mason bees most frequently visited mustard (4174 visits) and lacy phacelia (3950 visits) followed by buckwheat (1984 visits).

In cages with 100 honey bee individuals and 10–20 foraging honey bees on average 0.9 flower visits (of red mason bees and honey bees) per minute and per percent blossom cover were observed. In heathlands, we found the same number of flower visits per minute and per percent blossom cover (Hudewenz and Klein 2013). In cages with 300 honey bee individuals and 30–60 foraging honey bees, 1.8 flower visits per minute and per percent blossom cover were recorded.

**Figure 1:**
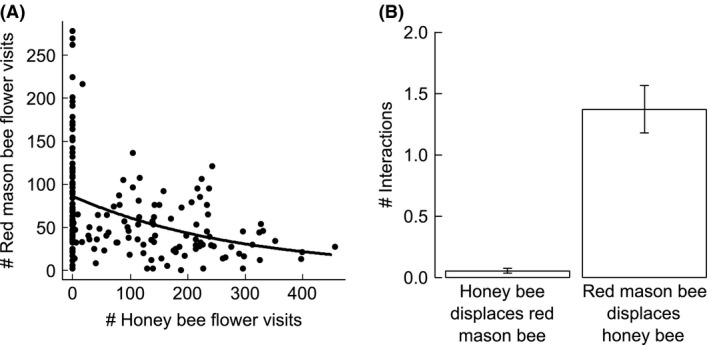
The relationship between the number of (A) red mason bee (*Osmia bicornis*) flower visits per observation interval (*n* = 168, df = 165) and the number of honey bee (*Apis mellifera*) flower visits per observation interval (*n* = 168, df = 165, GLMM, *P *<* *0.001) and (B) number of interactions (mean ± SE) per observation interval (*n* = 168) and honey bees and red mason bees.

As expected, the highest number of flower visits by honeybees was observed in cages with the highest density of honey bee individuals (300, Table 1, Fig. 1A). In cages with 300 honey bee individuals, we observed 21 ± 1 (mean ± SE) honey bee visits per minute and in cages with 100 honey bee individuals 7 ± 1 (mean ± SE) visits per minute. The number of observed flower visits by red mason bees decreased with increasing number of honey bee individuals (Hypothesis 1, Table 1, Fig. 1B) and with increased number of flower visits by honey bees (Table 1). In cages without honey bees, the red mason bees visited 10 ± 0.8 (mean ± SE) flowers per minute, in cages with 100 honey bee individuals 5 ± 0.3 (mean ± SE) flowers per minute, and in cages with 300 honey bee individuals 5 ± 0.5 (mean ± SE) flowers per minute. Fewer flower visits by red mason bees were observed when a high number of plant species was flowering during the observation (Table 1). Red mason bees (males only) were observed displacing honey bees from flowers 25 times more often than vice versa (Hypothesis 2, Fig. 1B, Video S1). In cages with 300 honey bees, red mason bees displaced honey bees 2 ± 0.4 (mean ± SE) times per observation interval (10 min), while honey bees displaced red mason bees 0.07 ± 0.04 (mean ± SE) times. In cages with 100 honey bees, we observed red mason bees displacing honey bees 1.7 ± 0.4 (mean ± SE) times per observation interval and honey bees displacing red mason bees 0.09 ± 0.04 (mean ± SE) times. However, red mason bee males were observed displacing conspecifics as often as displacing honey bees.

**Table 1:** Influence of certain explanatory variables on the number of flower visits of both species and the number of red mason bee brood cells. Results are from generalized linear mixed models

Response variable	Explanatory variable	Direction of response	*F*	*P*
Overall # honey bee flower visits	Honey bee treatment	+	152.9	<0.001
Overall # red mason bee flower visits	# Flowering plant species	−	12.14	<0.001
	Honey bee treatment	−	22.78	<0.001
	# Honey bee flower visits	−	33.42	<0.001
# Red mason bee brood cells	Honey bee treatment	−	16.9	<0.001

### Niche breadth and resource overlap

**Figure 2:**
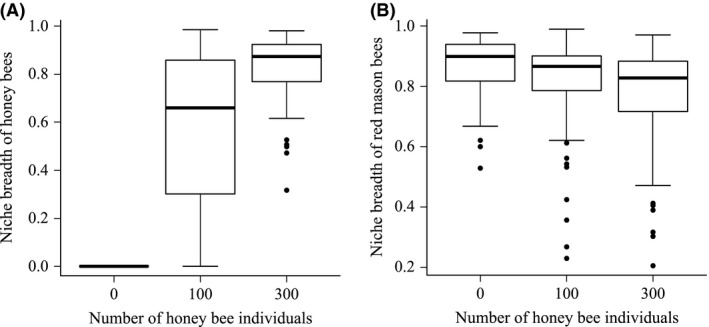
Niche breadths per observation interval of (A) honey bees (*n* = 168, df = 165, GLMM, *P *<* *0.001) and (B) red mason bees (*n* = 168, df = 165, GLMM, *P *<* *0.001) in cages without honey bees, with 100 and 300 honey bee individuals.

The niche breadth of honey bees significantly increased with increasing number of honey bee individuals (Hypothesis 3, Table 2, Fig. 2A). In cages with 100 honey bee individuals, honey bees had a niche breadth of 0.47 ± 0.28 (mean ± SD) and in cages with 300 honey bee individuals 0.69 ± 0.12 (mean ± SD). In contrast, the niche breadth of the red mason bee decreased with increasing honey bee individuals (Hypothesis 3, Table 2, Fig. 2B) and with increasing honey bee niche breadth (*F* = 3.97, *P *=* *0.048). In cages without honey bees, the niche breadth of red mason bees was 0.73 ± 0.1 (mean ± SD), in cages with 100 honey bee individuals 0.69 ± 0.16 (mean ± SD), and with 300 honey bee individuals 0.65 ± 0.19 (mean ± SD).

**Table 2:** Influence of honey bee treatment on the niche breadth of both species and their percentage overlap and Hurlbert's Index for resource overlap. Results are from ANOVAs

Response variable	Explanatory variable	Direction of response	*F*	*P*
Niche breadth honey bees	Honey bee treatment	+	210.5	<0.001
Niche breadth red mason bees	Honey bee treatment	−	7.316	<0.001
Percentage overlap	Honey bee treatment		0.819	0.368
Hurlbert's Index resource overlap	Honey bee treatment		2.136	0.147

Neither the percentage resource overlap nor the Hurlbert's Index for resource overlap was influenced by the number of honey bee individuals (Hypothesis 4, Table 2). The percentage overlaps in cages with 100 honey bee individuals amounted to 52 ± 5% (mean ± SE) and in cages with 300 honey bee individuals to 57 ± 3% (mean ± SE). In cages with 100 honey bee individuals, the Hurlbert's Index was 1.31 ± 0.16 (mean ± SE) and in cages with 300 honey bee individuals 2.01 ± 0.39 (mean ± SE).

### Reproductive success

**Figure 3:**
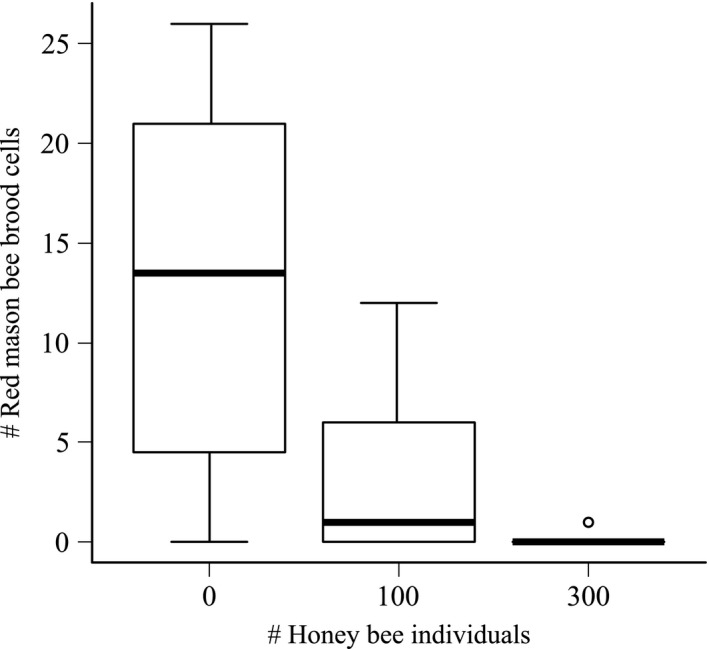
Number of red mason bee brood cells (*n* = 24, df = 21, GLMM, *P* < 0.001) in cages without honey bees, with 100 and with 300 honey bee individuals.

A total of 72 nests were counted comprising 131 brood cells. The females of the red mason bee built significantly more brood cells in cages without honey bees than in cages with 100 honey bee individuals or cages with 300 honey bee individuals, and the cages with the two different densities were also significantly different (Hypothesis 5, Table 1, Fig. 3). In cages without honey bees, the red mason bees built 13 ± 3.5 (mean ± SE) nests. In cages with 100 and 300 honey bee individuals, 3.3 ± 1.5 and 0.13 ± 0.13 (mean ± SE) nests, respectively, were found. This sums to a total of one nest in cages with 300 honey bee individuals.

## Discussion

The results of our study show that the red mason bee cannot compete with honey bees under densities which are common in open‐field conditions as in our cages with 100 honey bee individuals (Hudewenz and Klein 2013). We show that red mason bees visited fewer flowers when honey bees were present and the niche breadth of the red mason bee decreased with increasing honey bee density and niche breadth. Hence, the reduced number of flower visits by red mason bees likely is caused by honey bees occupying part of their niche space, a finding which has been confirmed by some field studies which also found reduced flower visits or abundances of wild bees with high densities of honey bees (Schaffer et al. 1983; Forup and Memmott 2005; Shavit et al. 2009; Hudewenz and Klein 2013) (although others did not find this correlation (Garibaldi et al. 2013)). Aizen and Feinsinger (1994) investigated the effect of habitat fragmentation on flower visitors and found that honey bees increasingly visited small flower patches, while wild bee visits decreased. On large flower patches, they found decreased honey bee visits but increased wild bee visits. This supports our findings that wild bee visits decrease when honey bees are present in small habitat patches where floral resources are limited.

Resource overlap did not differ between cages with 300 or 100 honey bee individuals. Both bee species are generalists that visit many different plant species. In our study, both species preferred the three most abundant flowering plant species, which explains the high degree of resource overlap. Compared to our study, Steffan‐Dewenter and Tscharntke (2000) found a slightly lower percentage of overlap of wild bees and honey bees but a higher Hurlbert's Index for resource overlap, which is likely due to differences in the plant species studied and the number of competing wild bee species.

Greenleaf and Kremen (2006) observed interactions between wild bees and honey bees in which wild bees displaced honey bees from flowers. We also found that males of the red mason bee displace honey bees more often than vice versa, with male mason bees displacing intraspecific males and females as often as honey bees. We did not observe females of the red mason bee displacing honey bees. Seidelmann (1999) observed males of the red mason bee inspecting other bee species, while searching for mating partners (females of the red mason bee) and thereby displaced the interspecifics from flowers. However, such effects may only occur temporarily depending on the season when high proportions of male bees are available. Thus, it seems not to be a competition strategy of the red mason bee to displace honey bees from flowers and prevent them from exploiting resources, but rather red mason bee males may be mistaking the honey bees for potential female conspecific mates.

The reproductive success of red mason bees was reduced in cages that contained high densities of honey bees. Also other studies found an influence of honey bees on the reproduction of wild bees (*Sugden and Pyke* 1991 *; Paini and Roberts* 2005 *)*. The reduced reproductive success might be caused by the lower number of flower visits and decreased niche breadth of the red mason bee, which thereby reduced the pollen supply provided by red mason bees to their larvae. Across all of the four cages with 300 honey bee individuals, we found only one red mason bee nest, which contained a single brood cell. The bee density from cages containing 300 honey bee individuals was higher than densities we found in open‐field conditions (Hudewenz et al. 2012; Hudewenz and Klein 2013), but such high densities might be reached temporarily before and after the peak flowering of crops or other mass‐flowering plant species or when introducing honey bee colonies into small and former honey bee free habitats. Our results show that under such high bee densities, the red mason bee population of such a small and isolated flowering patch could be displaced in the next year or the year after, especially when the start or end of peak crop flowering overlaps with the activity time span of a wild bee species. Many wild bees are active only for short periods of time, during which they need to find adequate resources to provision their offspring with food. Even in cages with 100 honey bee individuals (bee densities which we observed in open fields), we found a 75% decline in the reproductive success of red mason bees compared to cages with no honeybees. However, whether these effects persist outside of the cages, in open landscapes where wild bees can forage in higher distances, needs to be studied. The foraging distance of bees is related to their body size, such that those wild bees which are smaller than honey bees do not forage long distances (Gathmann and Tscharntke 2002; Greenleaf et al. 2007) and thus may not be able to avoid honey bees spatially. In contrast, honey bees are able to forage for distances up to several kilometers (Gathmann and Tscharntke 2002). Densities of honey bees are generally high in many open landscapes, like in our cages with 100 honey bee individuals, due to the presence of domesticated colonies, such that refugia for wild bees without honey bees are scarce.

## Conclusions

We conclude that the red mason bee cannot successfully compete with honey bees for flower resources in a controlled cage experiment. We encourage further controlled experiments to investigate the size of floral patches needed to avoid competition and natural field experiments to validate our results from controlled environments. These field experiments need to include ground‐ and stem‐nesting wild bees and, most challenging, need to use sites with and without honey bees foraging on flowers.

## Conflict of Interest

None declared.

## Supporting information


**Table S1.** Comparison of the ratio of wild bee/honey bee flower visits in the present study with two other studies that comprised flower observations.
**Table S2.** Target plant species of the “Tübinger Mischung” and proportions of their seeds to the total amount of seeds and non‐target plant species.

**Video S1.** This video shows a red mason bee male displacing a honey bee from a flower.
