# Supplementary material for: Red mason bees cannot compete with honey bees for floral resources in a cage experiment
Source: Ecol Evol. 2015 Oct 16;5(21):5049–56. doi: 10.1002/ece3.1762 (PMC4662317; doi:10.1002/ece3.1762)
Supplement: Supplementary file 1 — Table S1. Comparison of the ratio of wild bee/honey bee flower visits in the present study with two other studies that comprised flower observations. Table S2. Target plant species of the “Tübinger Mischung” and proportions of their seeds to the total amount of seeds and non‐target plant species. [file ECE3-5-5049-s001.docx]

Electronic supplementary material

Table S.1. Comparison of the ratio of wild bee/honey bee flower visits in the present study with two other studies that comprised flower observations.

| Study | # honey bee visits | # wild bee visits | ratio (# of honey bee visits to single wild bee visits) |
| --- | --- | --- | --- |
| Present study | 12,123 | 2,718 | 4.4 |
| Hudewenz and Klein 2013 | 2,762 | 84 | 32.9 |
| Hudewenz et al. 2012 | 569 | 221 | 2.6 |

Table S.2. Target plant species of the “Tübinger Mischung” and proportions of their seeds to the total amount of seeds and non-target plant species.

| Target plant species | Proportion (%) |
| --- | --- |
| *Phacelia tanacetifolia* Benth | 40 |
| *Fagopyrum esculentum* Moench | 25 |
| [Sinapis alba](http://de.wikipedia.org/w/index.php?title=Wei%C3%9Fer_Senf&oldid=27195655) L. | 7 |
| [Coriandrum sativum](http://de.wikipedia.org/w/index.php?title=Echter_Koriander&oldid=27042299) L. | 6 |
| [Calendula officinalis](http://de.wikipedia.org/w/index.php?title=Ringelblume&oldid=25008835) L. | 5 |
| [Nigella sativa](http://de.wikipedia.org/w/index.php?title=Echter_Schwarzk%C3%BCmmel&oldid=26802014) L. | 5 |
| [Raphanus sativus](http://de.wikipedia.org/w/index.php?title=Garten-Rettich&oldid=19532144) L. | 3 |
| [Centaurea cyanus](http://de.wikipedia.org/w/index.php?title=Kornblume&oldid=26965915) L. | 3 |
| [Malva sylvestris](http://de.wikipedia.org/w/index.php?title=Wilde_Malve&oldid=26798148) L. | 3 |
| [Antheum graveolens](http://de.wikipedia.org/w/index.php?title=Dill_%28Pflanze%29&oldid=25337968) L. | 2 |
| [*Borago officinalis*](http://de.wikipedia.org/w/index.php?title=Borretsch&oldid=24420270) L. | 1 |
| Non-target plant species | Density |
| [Chenopodium album](http://en.wikipedia.org/wiki/Chenopodium_album) L.  *Papaver rhoeas* L. | Low (< 2%) |
|  | Low (< 2%) |
| *Matricaria chamomilla* L. | Low (< 2%) |
| *Melilotus alba* Medic | 1 individual |
| *Viccia cracca* L. | 1 individual |
| *Geranium robertianum* L. | 1 individual |
| *Plantago lanceolata* L. | 1 individual |

Movie

Video S.1. This video shows a red mason bee male displacing a honey bee from a flower.
